# Atypical Presentation of Susac Syndrome in 55-Year-Old: From Unremarkable Stroke Workup to Rapid Diagnosis of “Snowball Strokes” and Successful Immunosuppressive Treatment

**DOI:** 10.1155/crnm/3178952

**Published:** 2025-04-27

**Authors:** Yasaman Pirahanchi

**Affiliations:** Neurology, Swedish Medical Center, Englewood, Colorado, USA

## Abstract

We report the case of a 55-year-old right-handed female with a medical history of hypothyroidism and gastric bypass surgery. The patient initially presented with cognitive impairment, dizziness, and unsteady gait. Despite an unremarkable stroke workup, her symptoms progressed rapidly within 2 days, leading to subsequent admissions and a complex diagnostic journey revealing Susac syndrome—a rare autoimmune disorder affecting the brain's microvasculature, retina, and cochlea. The patient's treatment involved aggressive immunosuppression with corticosteroids, IVIG, mycophenolate, and cyclophosphamide. The patient responded well and had progressive improvement, with discharge to home. This case highlights the diagnostic challenges and management strategies for Susac syndrome.

## 1. Introduction

Susac syndrome is a rare autoimmune disorder characterized by a triad of symptoms involving encephalopathy, monocular visual loss (due to underlying branch retinal artery occlusions), and hearing loss. The incidence of Susac syndrome ranges from 0.024 to 0.13 per 100,000 individuals [1]. It predominantly affects women aged 20–40 years but can occur at any age [2]. Although it is rare, the disease's hallmark radiological feature is corpus callosum involvement with lesions resembling “snowballs” or “holes” on brain MRI, often misdiagnosed initially as multiple sclerosis due to white matter disturbances [3]. In their review, Marrodan et al. highlighted the variable presentation of Susac syndrome, which results in significant underdiagnosis. These authors also concluded that Susac syndrome is often treated late, leading to severe and irreversible consequences such as dementia or vision and hearing loss [4]. Therefore, early recognition and aggressive immunosuppressive therapy are crucial to prevent irreversible damage and improve outcomes.

The pathophysiology of Susac syndrome is thought to involve autoimmune-mediated damage to the microvasculature of the brain, retina, and cochlea, with T cells and autoantibodies such as anti-GAD65 playing a possible role in disease progression. This autoimmune attack leads to endothelial cell damage and ischemic changes. There is also an apparent risk of recurrence. Petty et al. [5] described the case of a patient, age 31, who experienced significant symptoms, including memory, hearing, and vision loss, and responded well to corticosteroid treatments. Seventeen years later, the patient began taking estrogen and experienced a recurrence of visual disturbances, vertigo, balance issues, and hearing loss. Imaging revealed strokes along the corpus callosum, as seen on MRI FLAIR and T2 sequencing.

Wilf-Yarkoni et al. conducted a systematic review of recent articles in which ∼636 patient cases were reviewed. They found a mean age of onset of 29.5 years, a 57% white predominance, and a nearly two-fold higher prevalence in female patients. They examined 311 cases with available clinical presentation data at the onset of disease and showed that only a small proportion (37%) presented with the clinical triad. Most of the patients studied had central nervous system symptoms (68.5%), followed by ocular (61.1%) and neurological (51.8%) involvement, with headache being a common complaint (53%). Their findings support the importance of ophthalmologic evaluation along with the use of specific MRI sequences that demonstrate punctate white matter hyperintensities [6].

## 2. Case Presentation

**Figure 1:**
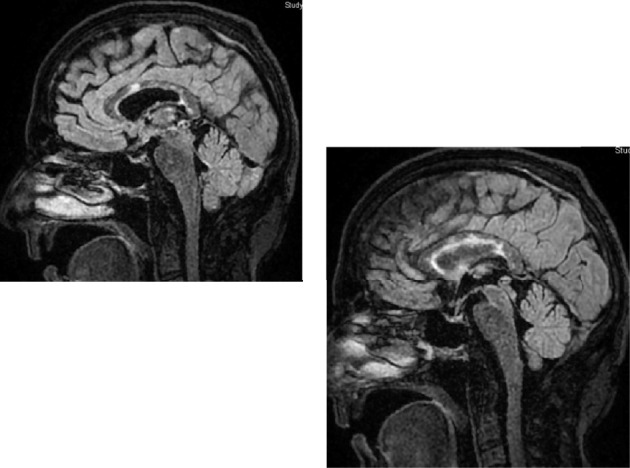
MRI findings with strokes along the body and splenium of the corpus collosum in the fluid attenuated inversion recovery (FLAIR) and T2 weighted MRI sequences, best viewed on sagittal sequences, with high signal regions that are well demarcated and spherical in shape, or referred to as “snowball strokes.”

A 55-year-old right-handed female with a medical history significant for hypothyroidism and gastric bypass surgery presented with a 1-2 weeks history of cognitive fog, dizziness, and an unsteady gait. Neurological examination was initially unremarkable, with a National Institutes of Health Stroke Scale (NIHSS) score of 0. Subsequent brain MRI revealed scattered acute/subacute infarcts involving the corpus callosum, suggesting a central embolic etiology. The stroke workup was negative, but her symptoms worsened rapidly within 1 week with progressive generalized weakness, flaccid bilateral lower extremities, and hyperreflexia upon re-admission. Susac syndrome was suspected based on MRI findings, which showed strokes along the body and splenium of the corpus callosum in the Fluid Attenuated Inversion Recovery (FLAIR) and T2-weighted MRI sequences. The MRI images revealed high signal regions that are well demarcated and spherical in shape, referred to as “snowball strokes” (Figure 1).

The diagnosis was further supported by cerebrospinal fluid (CSF) analysis, which showed pleocytosis with markedly elevated protein (WBC 8 cells/µL, protein 153 mg/dL, glucose 55 mg/dL). Normal vitamin levels ruled out potential nutritional deficiencies due to the patient's gastric bypass surgery. Aggressive immunosuppression was initiated with Methylprednisolone 1000 mg IV q24 h, followed by IVIG 0.4 g/kg/day for 5 days, and Rituximab 1000 mg for 2 days. The patient's condition improved, and she was transitioned to prednisone 80 mg/day and Mycophenolate 1000 mg BID. However, 14 days later, she presented again with confusion, regression of prior gait improvement, and repeat MRI showing additional new multifocal strokes.

She received 3 days of 1000 mg IV Methylprednisolone, followed by cyclophosphamide due to clinical progression. Fluorescein angiography showed evidence of bilateral branch retinal artery occlusions. The patient was placed on a prednisone taper from 80 mg/day to 40 mg/day over the course of 2 months, with a plan to continue Mycophenolate 1000 mg BID for 2 years. The patient showed further improvement with this treatment approach.

## 3. Discussion

This case underscores the diagnostic challenges of Susac syndrome, which can present with atypical features and progress rapidly despite an initial negative workup for stroke [7]. The patient's clinical deterioration and multifocal strokes underscore the importance of early recognition and prompt initiation of immunosuppressive therapy.

Management of Susac syndrome involves a combination of high-dose corticosteroids, IVIG, and immunosuppressive agents such as mycophenolate and cyclophosphamide [8]. For example, Kleffner et al. found that using formal criteria for diagnosing Susac syndrome, particularly through white matter lesions in the corpus callosum, can help optimize diagnosis. They also noted that relying solely on the clinical triad could lead to delays in diagnosis and treatment. Our patient presented with the triad of visual disturbances, psychiatric disturbances, and decreased hearing, and responded well to IVIG and corticosteroids, with discharge within 7 days. However, our patient also improved rapidly upon treatment, which is not always the case, and close monitoring for disease progression and potential complications, such as retinal artery occlusions, is essential for optimizing outcomes [4, 9].

Wilf-Yarkoni et al. [6] and Bose et al.'s [10] systematic reviews revealed the complexities of identifying Susac syndrome and recommended using IVIG and corticosteroids for treatment. We followed these treatment guidelines, and the patient rapidly improved in visual, psychiatric, and hearing symptoms. However, variations in clinical presentation should be considered, as the triad is not necessarily diagnostic in every case. Wilf-Yarkoni's work suggests that prompt ophthalmologic evaluation and MRI sequences demonstrating punctate white matter hyperintensities are critical diagnostic components.

## 4. Conclusion

Susac syndrome is a rare and complex disorder that requires early recognition for effective treatment. While the clinical triad is a useful diagnostic tool, the disease can present with atypical features, making early and comprehensive evaluation essential. This case highlights the importance of considering Susac syndrome in patients with unexplained neurological symptoms, even when initial diagnostic workups are unremarkable.

## Data Availability

The data that support the findings of this study are available from the corresponding author upon reasonable request.
